# The role of context and the interpersonal experience of loneliness among older people in a residential care facility

**DOI:** 10.3402/gha.v5i0.18861

**Published:** 2012-10-11

**Authors:** Vera Roos, Lelanie Malan

**Affiliations:** 1 Psychology, School of Psychosocial Behavioural Sciences, North-West University, Potchefstroom, South Africa; 2 4 Little Swift Street, Van der Hoffpark, Potchefstroom, Noordbrug, South Africa

**Keywords:** context, coping with loneliness, crystallization, emotional loneliness, interpersonal experiences, Mmogo-method^®^, older people, residential care facility, social isolation

## Abstract

Older people are more prone to experience loneliness when living in residential care facilities. The purpose of this study was to explore older people's experiences of loneliness in the context of institutionalized care. A voluntary and convenience-based sample of 10 white South African older people (age range 62 to 82 years; three men and seven women) was drawn. Data on the subjective experience of loneliness were then gathered through the Mmogo-method^®^, whereby drawings were employed to explore matters and issues of importance in the lives of older people that could be used to deal with loneliness. Data were analyzed thematically and visually as well as through the use of keywords in context. The results showed that older people experienced loneliness in terms of having unavailable interactions due to loss, and an absence of meaningful interpersonal interactions. Meaningful interpersonal interactions were described as when the older people had regular contact and a variety of interactions. Ineffective interpersonal styles (e.g. taking a controlling position in relationships and being rigid) elicited rejection and isolation, and were associated with a lack of confirmatory interpersonal relationships. It is recommended that greater emphasis should be placed on creating awareness of unhealthy group dynamics as well as on psychosocial interventions to develop group support. Interpersonal styles, either effective or ineffective, take place in a social context, which, in this research, was observed to be unsafe, lacking in care, and a non-stimulating environment.

Loneliness is a global phenomenon among an increasing older population, people older than 60 years of age. It is estimated that there were about 3.8 million older people in South Africa in 2010 (1). This segment of the population constituted roughly 7.6% of the total population at the time (1). Furthermore, many older people have to be looked after in residential care facilities owing to their deteriorating health, the migration of children and grandchildren, and their limited financial resources. Older people placed in residential care facilities often experience loneliness (2).

Loneliness has significant implications for mental health as well as cost implications for government. Booth (3) states that loneliness is a condition with various problematic implications for mental health, including feelings of sadness, a sense of uselessness, and an inability to interact with other people. As reported by some researchers, a link exists between the health status of individuals and experienced loneliness (4). Loneliness can accelerate the breakdown of a person's health status because of its effect on the body's immune system, which, in turn, impacts on an individual's mental and physical health (5). The growing ageing population nationally and internationally places greater demands on the financial resources of governments, and it is therefore important to invest in preventative interventions.

Loneliness is generally closely associated with ageing as a consequence of multiple losses – loss of abilities, loss of and changes in personal relationships, loss of relationships with familiar environments, and changed contact with friends and relatives resulting in reduced relationships (6–8). Changes in the relationship with the environment are regarded as a particular loss by older people, especially if they have to rely on institutionalized care and lose contact with familiar social networks and free association with other people (8).

Loneliness, according to Sullivan (9), is an ‘exceedingly unpleasant and driving experience connected with inadequate discharge of the need for human intimacy’ (p. 290). Much research has focused on the subjective experiences of loneliness (10–13); however, the way loneliness manifests in an interpersonal context is not clear and requires further investigation. The research question that guided the present study was therefore: What are the perceptions of older people about loneliness, and what things do they regard as important in their lives which can be harnessed to deal with loneliness?

Lonely people are isolated people. Weiss (14) distinguishes between two types of isolation – social isolation and emotional isolation. Both types of isolation refer to interpersonal interactions with individuals. Emotional isolation is associated with the loss or the absence of a significant person or partner, unsatisfactory child–parent relationships, and the absence of intimate friendships. Emotional support is often limited, feelings of sadness and longing are expressed, and the need for closeness with significant others is frequently frustrated (10, 11). Social isolation, experienced as boredom and insignificance, occurs when people feel that they do not belong and that they do not have meaningful connections with supportive networks (10, 11, 14). Loneliness is defined as the subjective experience of discomfort due to inadequate social exposure and experience, ineffective interpersonal interactions, and the lack of social interactions due to relocation or due to the death of significant others (12).

For the purposes of this study, loneliness was regarded as a relational phenomenon, and the theory of complex responsive processes of relating was used as the study's theoretical framework (15). People communicate continuously, both in their private conversations with themselves as well as by interacting with others, which refers to public conversations (16). Suchman (15) maintains that the gestures and responses in public conversations originate from people's private conversations and vice versa. Thus, the way in which the private conversations within a person are constructed influences the perceived public conversations that people have with one another. Certain limitations and conflicts within the private conversations of people can deter them from taking part in public conversations. The aim of this study was therefore to explore older people's experiences of loneliness in a residential care facility in respect of what they regarded as important in their lives.

## Research method and design

An exploratory research method was used in an attempt to understand the interpersonal experiences associated with loneliness (17). Qualitative research methods enable participant-generated meanings to be heard in conversations or text (18, 19). Evidence gathered through qualitative methods can be used to explain the meaning that older persons attach to their experiences of loneliness (17, 20, 21).

## Research context and participants

The research was conducted in a residential care facility in Johannesburg in the Gauteng Province of South Africa. The facility can be described as an economically deprived residential care facility with limited health and financial resources. The facility serves frail older people as well as independently functioning older people. The residents have to share accommodation, and few opportunities exist to engage in group activities following the termination of many activities due to lack of interest.

Participants were invited to participate in the research through the management of the residential care facility and the participants indicated their willingness to participate. The voluntary group of older people was cognitively able to participate in the research activities, to communicate in either English or Afrikaans, and was living in a residential care facility. The group consisted of 10 older persons (seven women and three men) with ages ranging between 62 and 82 years. Seven of the participants were widowed, one was single, one was divorced, and one was married. Most of the participants had an average of two children with only two of the participants not having any children. Nine of the participants were dependent on their pension funds with only one participant being supported by relatives. The participants mentioned participating in various activities on their own, including painting, photography, singing, needlework, knitting, reading, sewing, and listening to music. Only one participant indicated that he did not participate in any activities on his own. It was also observed that only three participants were involved in community organizations such as the SAVF (South African Women's Federation) – the remainder indicated that they had no involvement with community or welfare organizations.

## Data gathering and procedure

The social worker at the residential care facility requested an assessment of the experiences of loneliness of the residents. On the day of the data collection, the participants were informed about the objectives of the research and their involvement. They were also told that they would be required to make visual representations of their experiences of loneliness using a visual projective data-gathering instrument, the Mmogo-method^®^ (22). The Mmogo-method^®^ uses the subjective nature of people's frame of reference to obtain insight into their subjective lived experience as well as their relationships (22). With the Mmogo-method^®^, the participants would use clay, beads, and sticks to construct their visual representations based on an unstructured request: *Please make a visual representation with the materials provided that can tell us more about how you experience loneliness*. The Mmogo-method^®^ is used by social researchers to gain a deeper understanding of people's social life experiences, perceptions, and behavior (23). In the Mmogo-method^®^, participants sit around a table where they construct their visual representations simultaneously. After all the participants have completed their representations, they talk about what they have made and the meaning of their representations. These discussions provide the researcher with additional information and the opportunity to verify the data.

In a second data-gathering method, the participants were requested to draw visual images of anything in their lives that they regarded as important (24). In response to the request, *Please make a visual representation of yourself and anything of importance in your life by using circles*, the participants had to use paper and a pencil to draw circles representing themselves and people or things of importance. The unstructured formulation of the request was important as it gave the participants the opportunity to respond openly and to explore the things of importance in their lives without restrictions. The drawings were shared with the other participants, and explorative questions were asked by the researchers to gain a better understanding of the participants’ drawings, such as: (1) What do the circles represent for you? and (2) Can the important things that you have indicated in the circles be used to help you deal with your loneliness? Please give reasons for your answers. According to Theron et al. (24), one can explore the experiences of people regarding a specific phenomenon using picture drawing as a projective technique in such a way that conscious and unconscious issues arise. In this way, more descriptive information can be obtained.

## Data analysis

The collected data were transcribed and analyzed using thematic analysis and key-words-in-context analysis, which contributed to the trustworthiness of the research. The visual presentations and drawings were analyzed by means of visual analysis.

### Thematic analysis

The thematic analysis consisted of the analysis of data sets in six phases (25) in terms of which the data were read and reread and categorized into broad themes (26). The first step in data analysis always involves familiarization with the data (25), which includes reading and rereading the data as well as noting key ideas. The second step involves generating initial codes where features of the data are identified through the codes (25). Henning (27) states that qualitative coding means that the data are divided into small units of meaning and then grouped into categories. The third step involves the search for themes. The themes are driven more by the data than by theory (25). Terreblanche, Durrheim, and Painter (28) argue that researchers should try to move beyond merely summarizing content—they should also organize principles that naturally underlie the material. In addition, they should analyze the different codes and consider how the codes could be combined to form an overreaching theme. During this phase, the visual representations are used to sort the different codes, which are presented through a mind-map, into themes. The fourth step involves reviewing and refining the themes. The themes should link together meaningfully so that they can be identified and distinguished from each other. The fifth step involves defining and naming the themes. It is important to identify the essence of each theme and to determine the specific aspect of the data each theme captures (25).

### Visual analysis

In the study, the researchers also visually analyzed the visual representations and drawings. According to Roos (23), visual representations reflect the conscious meaning that participants project about a particular phenomenon. The researchers analyzed the visual representations by observing what specific objects the participants made; the relationship between the objects such as the distance between the objects; the actions in which the objects are involved; the relational context as well as the broader social–cultural–political environment in which the objects are placed; and how the objects related to the research question. During the analysis of the drawings, the researchers paid attention to how many circles the participants drew, as well as the distance between the circles and the objects in the circles. Finally, the themes generated by the visual analysis of the representations were linked with themes identified through the other methods of analysis, thereby ensuring the trustworthiness of the data.

### Key-words-in-context method

The descriptions of loneliness in terms of its interpersonal features were analyzed using the key-words-in context method (KWIC). KWIC refers to viewing words that are of interest to the researcher by identifying the words used before and after a key phrase (29). The key phrases used in this study were loneliness and interpersonal relationships, for example, ‘… this was the beginning of my loneliness’ and ‘… the children, they don't want me anymore’. These words were then interpreted within the specific context of the research project rather than interpreted separately. The transcribed data were read, and keywords in phrases that occurred regularly or in a strange manner in relation to the interpersonal experiences of loneliness were identified. Leech and Onwuegbuzie (29) argue that such identified words or phrases should be listed in table format. The words that fit with the particular phrases are noted on the left and right side of the phrase as formulated in the original text. An interpretation of the words is written on the right side of the columns. This interpretation is compared with the interpretations of other phrases to identify underlying relations and recursive relationships between the concepts to make sure that a phrase illustrates the most intended meaning. Together with thematic content analysis, this method helps ensure the trustworthiness of the study.

Through crystallization, the researcher ensures that the clearest possible picture of the research topic has been constructed (27). Crystallization is a methodological framework for bringing together different forms of data and analysis as well as different genres and forms of sense making within interpretive methodology. This was achieved in the present study by analyzing the data, using three methods of data analysis, and reporting the results using the visual representations and drawings of the participants. This process facilitated the construction of a more detailed and rich representation of the phenomenon under investigation (30). Underlying meanings in different data sources can generally be discovered through crystallization as crystallization allows the interweaving and blending of data resulting in a more descriptive understanding of the research.

The researchers used two different qualitative data-gathering methods, namely the Mmogo-method^®^ and drawings, which provided a descriptive explanation of how the older people in the study experienced loneliness and how they interacted in social relationships and networks. The study also made use of various qualitative analysis methods including the KWIC method, thematic analysis, and visual analysis to obtain a clearer understanding of the research phenomenon. The researchers took steps during the research process to ensure that the findings were not romanticized or biased. They also, through constant reflection, endeavored to eliminate subjective perceptions and preconceived ideas regarding the participants and their experiences of loneliness. Images were included in the research report to provide a more descriptive understanding of the research topic, thereby helping ensure the trustworthiness of the study.

Ethical approval for the research project was obtained from the North-West University, ethical number (NWU-00053-10-S1). The guidelines as laid down by the Health Professions Council of South Africa for Psychologists (Health Professions Act 56 of 1974) were followed during the study. Ethical approval from the participants was obtained through their written consent. The participants were informed about the aims of the research project; what were expected from them; what the data were used for; the termination of their participation in the study; confidentiality; and the safekeeping of records, material, and recordings. All records, material, and recordings were treated as private and confidential and kept safe by the North**-**West University. The participants were told about the various phases of the research project and their right to terminate their participation at any time for any reason.

## Results

The data revealed that the older people in the study experienced loneliness when they could not participate in activities that had been important to them previously. The importance of the loss of activities in residential care facilities is widely confirmed by the literature (8). The contribution of the study to the body of knowledge on the elderly is that it casts further light on the interpersonal experiences that older people associate with loneliness. In this regard, two main themes with subthemes emerged. In theme one, relationships in the residential care facility were described in terms of (1) their experiences of the interactions in relationships and (2) preferred interpersonal styles. In theme two, the relationships in the context of the residential care facility were described as (1) unsafe and (2) lacking in care while the (3) environment was described as non-stimulating. The table below summarizes the themes and subthemes that emerged from the data analysis (Table 1).

**Table 1:** Themes and subthemes of the participants’ experiences of loneliness

Themes Experiences of relationships	Subthemes	

	Interactions in relationships	Interpersonal styles
	Unavailable interpersonal interactions Loss of meaningful interpersonal interactions ‘A grave. It was at a grave like this where my loneliness and life alone started.’ Absence of current meaningful interpersonal interactions Meaningful interpersonal interactions Regular contact ‘You know … in my room there are always people, so I am never alone.’ Variety of interactions	Ineffective interpersonal Styles ‘They don’t want me anymore. It feels like I don’t exist to them anymore.’ Effective interpersonal styles ‘There is nothing in my life that has happened that she doesn’t know about … she stood by me through sickness … she stands by you … the great thing about her is that she doesn’t talk out.’
Relationships in context	Unsafe environment ‘Confidentiality is non-existing, it’s non-existing, because before we know something, they know it.’ Environment lacking in care ‘You talk to a person and the person hears you, but he doesn’t really understand what you mean.’ Non-stimulating environment ‘Time, there is too much time on your hands … you can’t do anything … there is nothing to do …’	

### Experiences of relationships

The experiences of relationships were associated with the interactions in relationships as well as with the interpersonal styles.

#### Interactions in relationships

Interpersonal relationships were generally described in terms of unavailable interactions due to the loss of past meaningful interpersonal interactions as well as the absence of current meaningful interpersonal interactions. Meaningful interpersonal interactions were described as having regular contact and different interactions with others.

#### Unavailable interpersonal interactions

Interpersonal interactions were unavailable due to the loss of meaningful relationships in which the interactions took place as well as the absence of current meaningful interpersonal interactions.

*Loss of meaningful interpersonal interactions.* Almost 40% of the participants reported that they had lost meaningful interpersonal interactions through death. This specific loss was identified as the main reason for their loneliness. The following visual representation shows a grave made by a participant. It represents the time her loneliness began and according to her, ‘A grave. It was at a grave like this where my loneliness and life alone started’.

**Fig. 1:**
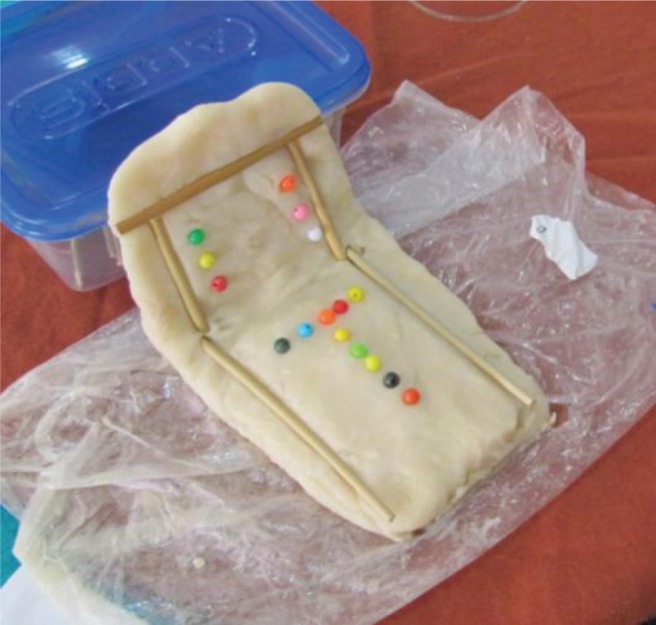
A grave illustrating the onset of loneliness.

Fig. 1 illustrates the participant's experience of losing a meaningful interpersonal interaction.

The participant mentioned that she lost both her husband and son through death and that was when her loneliness started. The loss of meaningful interpersonal interactions for the participants was accompanied by feelings of longing, frustration, and depression. They expressed feelings of longing for their family and friends who were either living far away or had died. Some of the participants mentioned that they longed for their earlier life because then they had more friends and relatives: ‘The whole way of living before … you had more friends, more family. Another participant responded, ‘Give me back the past … I was happy then, beautiful, family’. One participant mentioned feeling frustrated and depressed at times because she no longer had as much contact with her family: ‘I also get very lonely because I miss my children terribly, they can't visit me because they're in Port Elizabeth and I get very frustrated and depressed at times’.

*Absence of current meaningful interpersonal interactions.* Three participants reported not having anybody to talk to about their feelings and experiences of loneliness in their current interpersonal context. One said, ‘It makes you lonely, because you can't always communicate like you want to … you can't tell other people about how lonely you are’. Another participant stated that she experienced loneliness ‘when there is no one to take away your attention’, and another added, ‘If one has the need to talk to somebody and you go into that guy's room at 10 o'clock at night, then he will chase you away’.

#### Meaningful interpersonal interactions

In the context of the research study, meaningful interpersonal interactions refer to the participants having regular contact with others as well as having a variety of interactions within their personal environment including interaction with a Divine Reality, relatives, and friends.

*Regular contact.* The participants who reported having regular contact with other people said that they did not experience loneliness. One participant said, ‘I can't be lonely, because I always have people around me, I actually don't have enough chairs’. And another added, ‘You know … in my room there are always people, so I am never alone’. One participant mentioned having regular contact with other residents, ‘Luckily I can still move … I do the people's shopping for them, I do all kinds of things for them, and I enjoy it’.

**Fig. 2:**
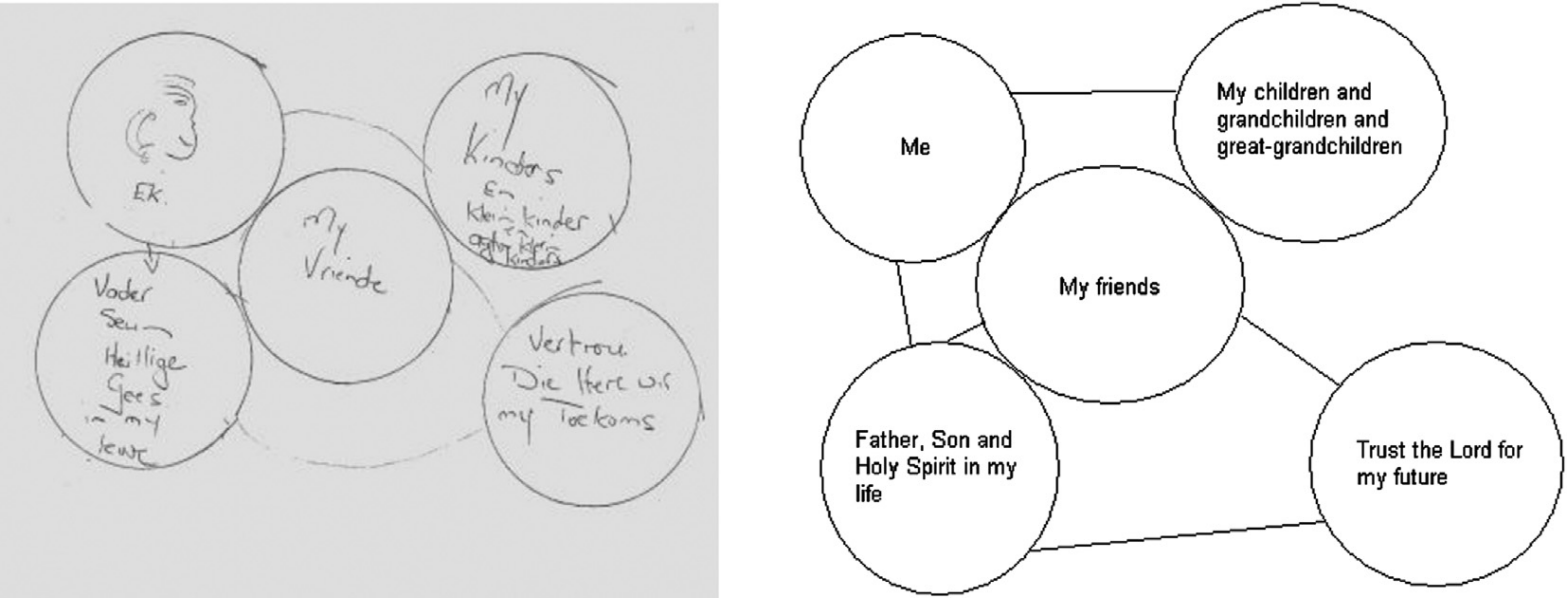
Drawing that illustrates meaningful relationships.

*Variety of interactions.* The participants described having various interactions in the course of their daily life. One participant talked about her interaction with a Divine Reality, which probably referred to her close relationship with God or a Supreme Being: ‘This is just me and the Father, Son, and Holy Ghost in my life. I trust the Lord for my future’. She drew an arrow from herself directly toward her Divine Reality, thus indicating the importance to her of the relationship in terms of comfort and support as can be seen below in Fig. 2. The particular participant also shows her close relationship with her children in Fig. 2 by drawing an arrow toward them and thus connecting herself directly with them. She also drew a circle with her friends in close proximity to herself, barely leaving any space. This could be part of the variety of meaningful interpersonal interactions the participant mentioned.

Fig. 2 illustrates the participant's comforting relationship with her Divine Reality, her friends, and her family.

#### Interpersonal styles

Interpersonal styles refer to the way in which people interact with other people (32). The data revealed two subthemes underlying interpersonal styles, namely ineffective interpersonal styles and effective interpersonal styles.

#### Ineffective interpersonal styles: rejection and worthless

**Fig. 3:**
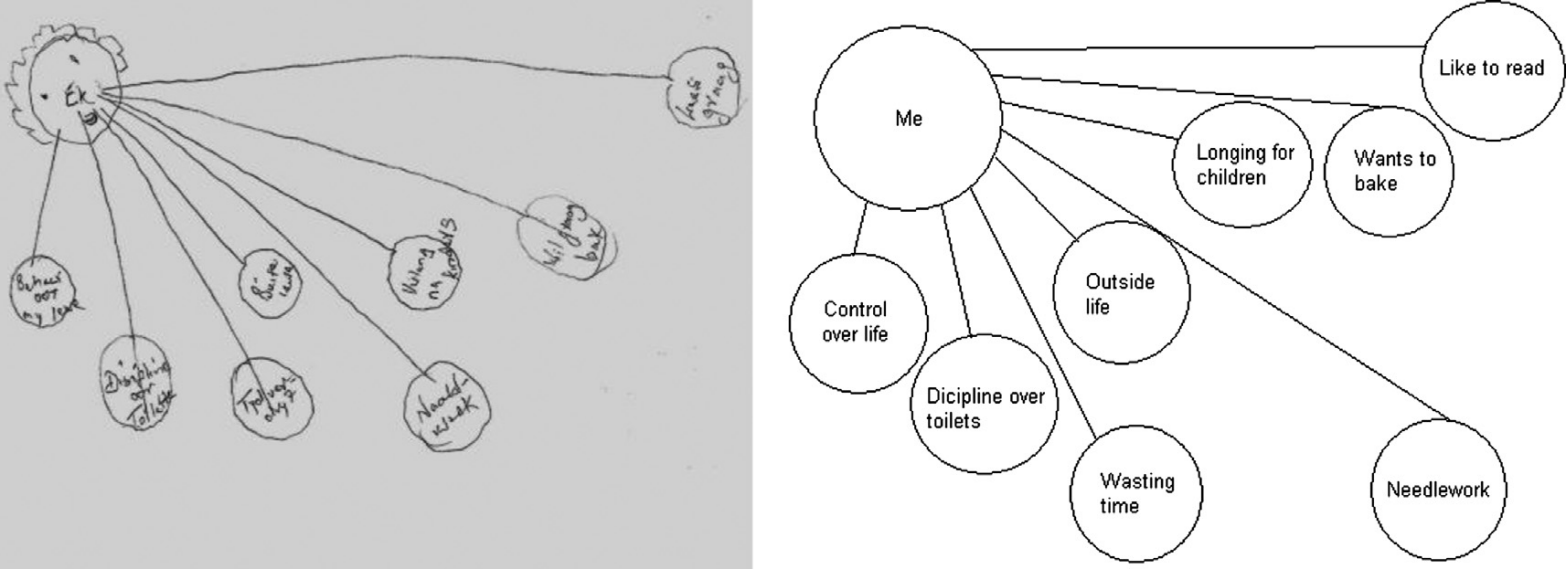
Drawing that illustrates the distance between important things and people and the self.

Ineffective interpersonal styles refer to an individual not having the personal repertoire to communicate effectively with other individuals in a way that facilitates the formation of meaningful interpersonal relationships. One participant said that she insisted on doing things only her way, ‘I am a strict grandma. I am not going to turn around, a thing should be right, otherwise grandma is strict’, and she added, ‘… control that I had …’. This participant described her controlling position in her relationship with her children and grandchildren. Her inability to adopt a more flexible interpersonal style contributed to an interpersonal environment in which she experienced isolation and possible rejection from other people, especially her children and grandchildren. She indicated considerable distance between herself and her family by drawing herself in a corner and her family far away from her. It could be said that her unwillingness to change and her continuous need to control others distanced her from everything important to her, especially her family. Fig. 3 illustrates the participant's experiences of her interpersonal relationships.

**Fig. 4:**
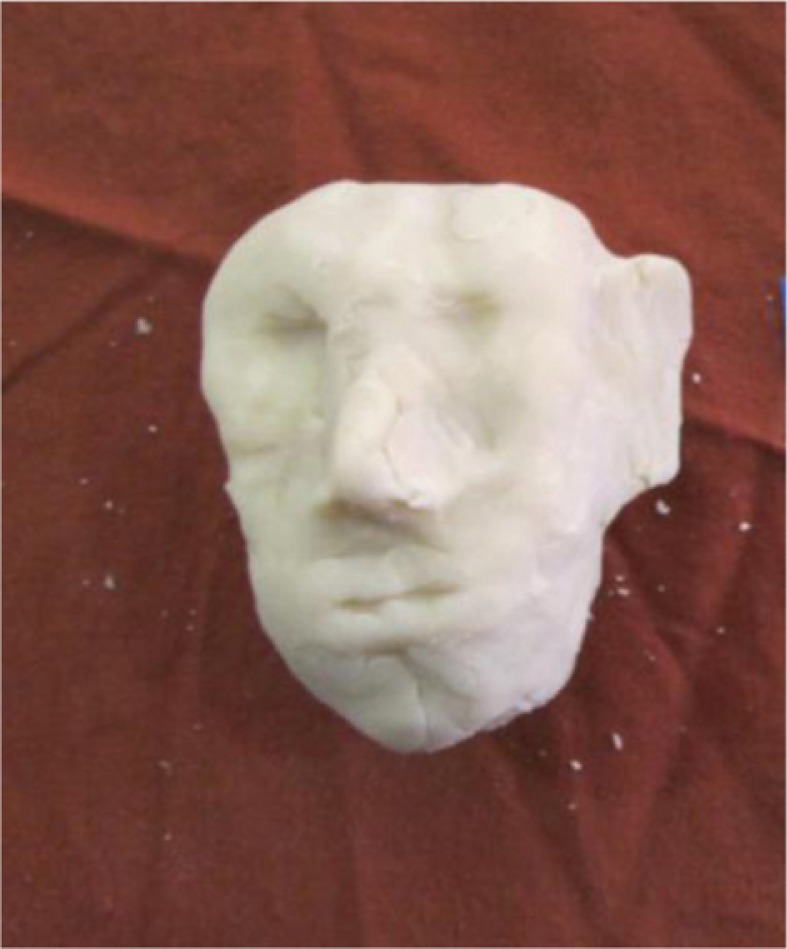
Only a face to the family.

Another participant indicated through his model that he existed for his children only as a face and nothing more. He said that he was such a difficult and cruel person his children avoided making contact with him: ‘I am a difficult person, you can say, a cruel person … I always just wanted my own way and all those things. Hurt everybody’. The participant also expressed feelings of rejection and worthlessness: ‘They don't want me anymore. It feels like I don't exist to them anymore’. Fig. 4 illustrates the participant feeling only like a face, a shadow.

#### Effective interpersonal styles

Effective interpersonal styles refer to the ability to form meaningful and supporting interpersonal relationships with others. In such relationships, one can become transparent, be respected, and receive confirmation from others. One participant mentioned having a confidant in the residential care facility with whom she had an interpersonal relationship characterized by support, trust, and feeling valued. ‘Yes, M is important to me. I can talk to her about things, because I know she understands. She knows me … she knows about everything, she knows about that pain’.

In respect of encouraging interpersonal relationships, only a few of the participants said that interpersonal relationships with friends provided them with encouragement, comfort, and support. One participant mentioned having a friend, not currently living with her in the care facility, with whom she shared everything that had happened in her life: ‘There is nothing in my life that has happened that she doesn't know about … she stood by me through sickness … she stands by you … the great thing about her is that she doesn't talk out. You can open up your little heart towards her … she doesn't go to the next person and talk about it … that is the most important.’ According to the participant, having a meaningful interpersonal relationship with a friend meant she felt safe sharing her deepest feelings and thoughts with the friend because she could trust her.

### Relationships in context

The relationships in the residential care facility are described as unsafe, lacking in care, and the environment as non-stimulating.

#### Unsafe interpersonal context

The interpersonal context in the residential care facility was described as unsafe for forming interpersonal relationships. The experience of the residents was that sensitive information about them was not treated with confidentiality but shared with others in the facility. Seemingly the managers of the residential care facility were targeted by the residents to share confidential information of fellow-residents in an attempt to draw attention from the managers. All the participants expressed a need for trust and respect in the institutional care facility so that supportive interpersonal relationships could be established with one another.

One participant responded as follows: ‘It is in a great sense … to deal with something. If I come and talk to you, then I open up my heart to you. It helps me to talk, but it is not going to help me to hear that you went to another and made up a whole new story. That is what hurts and damages self-worth the most …’. Most of the participants referred to the need for mutual respect in the care facility. They said the following when they heard stories about one another: ‘But there's only a few [people] that maintain a secret in this place’ and ‘We compare what we heard yesterday, to today … and it's getting better’. The older people showed little concern for the feelings of others and little respect for the stories shared by others, which was becoming a major problem because ‘they have nothing else to do … off course they will gossip’.

The issue of confidentiality arose because the older people apparently did not respect one another enough to guard sensitive information shared with them; instead, they used it as a way of drawing attention to themselves: ‘They can't live without it … it is actually for them something like a prestige … because they can talk about that one and tell something about that one’. This was also the case when the residents shared confidential information about fellow residents with the management of the care facility: ‘Confidentiality is non-existing, it's non-existing, because before we know something, they know it. And everybody knows it … and the security at the gate will know it …’ because ‘it is a basic disrespect … one needs respect and trust … respect means a lot’, as one participant reported. Finally, the data revealed that the divulging of confidential information about fellow residents made it difficult for the older people to form trusting interpersonal relationships with one another.

#### Lacking interpersonal care

Most of the participants experienced a lack of care from other residents – they felt they were not heard or understood by others: ‘If one has the need to talk to somebody and you go into that guy's room at 10 o'clock at night, then he will chase you away’ and ‘You talk to a person and the person hears you, but he doesn't really understand what you mean’ and ‘… mmm … I mean they can listen, but don't hear’. One participant said that other people were not interested in hearing about your life, ‘I mean, if only they were interested’ this could lead to ‘being really intimate’ but, in reality, ‘they don't hear, they only listen’. The participants thus expressed a need for intimate and trusting relationships, but the lack of a care environment in the facility prevented them from entering into such relationships: ‘You are not going to talk to someone that is careless about how you feel…’.

#### Non-stimulating environment

There were insufficient organized activities for the residents: ‘I mean in other places you get a dart board and sort of a game you can play, there is nothing here … I use to sing in operetta and choir’. One resident (participant) mentioned that she participated in her own individual activities when she was alone in her room, which was generally the case, to keep herself busy: ‘I keep myself busy with needlework. Any needlework, machine work, hackle work and knitting work’.

**Fig. 5:**
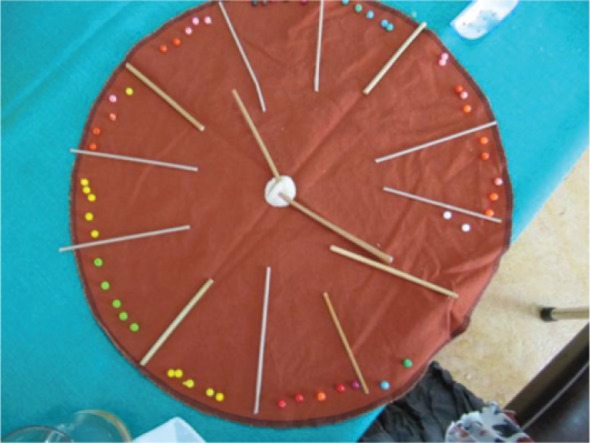
Clock illustrating too much time available.

However, the non-stimulating environment can also refer to participants not being introduced by more activities within the institutional care facility, causing them to feel bored. The participants’ boredom could possibly contribute to the ‘unsafe’ environment as the residents often spread stories in order to stay busy as previously mentioned by a participant. One participant said, ‘Time, there is too much time on your hands … you can't do anything … there is nothing to do …’. Fig. 5 illustrates the participant's experience of having too much time and not enough activities to stimulate him.

Having too much time and insufficient stimulating activities was an experience shared by most of the participants, accompanied by intense feelings of boredom and entrapment. According to one participant, ‘Me, in a little house, or in a room. Locked up in a little room … All I do is eat and sleep’. Another reported, ‘I am used to quite a variety of things I should do, sing and art and that type of thing, which I can't do here … I can't do anything about it. I'm actually stuck doing nothing … you can't get out. So it is a thing of eating and going to your room, you just lie on the bed…’. The participants’ experiences of a non-stimulating environment meant they were not exposed to activities that could facilitate the establishment of interpersonal relationships.

## Implications of the findings

Loneliness was subjectively experienced as being the result of the loss of previous meaningful interactions and the current unavailability of meaningful interactions. Loneliness as a consequence of the experiences of loss is not unique to the older persons in the present study. The literature confirms that old age is associated with numerous losses that are linked to experiences of loneliness (14, 31). Loneliness was expressed and described by the participants in terms of interpersonal relationships.

The experiences of relationships that were currently unavailable seemingly emerged from the older people's unwillingness to engage with one another as well as their general inability to participate in meaningful relationships. The inability to engage in meaningful relationships seemed to be related to the preferred interpersonal styles of some of the older people in the residential care facility in their relationships with other people. The interpersonal style of people who interact with other people in a controlling manner and who give and receive no empathy or positive acceptance from other people was confirmed by literature (32). Their rigid manner of engaging with others elicits rejection. In their reciprocal interactions with other people, their need for recognition and confirmation remains unfulfilled, and ultimately they become isolated individuals. The isolation is then experienced in relation to peers in their close social network. The literature indicates that older people depend on the support of members of their social network (34).

Conversely, the older people who indicated that they experienced meaningful interpersonal relationships described regular contact and a variety of interactions with other people. Roos and Klopper (8) and Townsend (35) emphasize the importance of engaging with others as an effective way of coping with loneliness. Key relational qualities in meaningful relationships are empathy for other people, confirmation of other people, and the ability to express their needs effectively and to make themselves more visible in the interpersonal contact.

The interpersonal context in which the interactions in the residential care facility took place was described as unsafe, lacking in care, and non-stimulating. Watzlawick, Bavelas, and Jackson (36) and Allport (37) stress the importance of the context in establishing meaningful and trusting interpersonal relationships. People feel emotionally unsafe when they experience a lack of confidentiality concerning their personal information. Evidently, some of the older people used confidential information to gain the attention of the manager of the care facility. The acquisition of prestige by divulging confidential information about others to authority figures is in line with social identity theory, as confirmed by Duckitt (38). Consequently, the people in the care facility experienced violations of their trust. The implications are that even if the older people had the capacity to establish meaningful interpersonal relationships, the environment in the facility appeared to be too threatening and unfavorable for meaningful relational interactions to take place. Baron and Byrne (39) confirm that more contact between older people in residential care facilities can help reduce preconceptions and disrespect if the contact occurs under favorable conditions. Emotionally, the environment in the particular residential care facility was perceived to be too dangerous for the older people to risk becoming visible to each other and to establish meaningful interpersonal relationships. Coalitions were formed to deal with the relational distrust, and those who were not included experienced isolation and loneliness. De Wet (40) confirms the formation of coalitions to satisfy specific needs within a group. The environment in the residential care facility lacked stimulating group activities, which provided fertile ground for boredom.

## Limitations and recommendations

The findings of the study are limited to a group of white older people living in Johannesburg, South Africa, and cannot be generalized throughout the population of older people living in residential care facilities. Managers of residential care facilities should be made aware of their role in creating a safe environment by not acting on confidential information provided by residents. Managers of institutional care facilities should also be made aware that older people will approach them with confidential information about other residents in an attempt to receive recognition and acceptance. Older people in residential care facilities should be made aware, through group work and intervention, of their interpersonal styles and the impact these styles have on the forming of trusting and meaningful interpersonal relationships.

In addition, more awareness should be created about the impact of breaking confidentiality on sensitive and private information as well as the effect of people's lack of care toward one another. More emphasis should be placed on enabling older people to take care of each other by respecting confidential information shared with one another, by respecting other residents, and by focusing on the establishment of trusting interpersonal relationships. Managers of residential care facilities should be made aware of the importance of making group activities available to older people as a means of enabling them to establish meaningful and trusting interpersonal relationships with one another.

## Conclusion

The older people in the study expressed loneliness in terms of the interpersonal experiences. The older people reported that they experienced loneliness when they lost previous important relationships and when they were not able to establish meaningful relationships in the facility environment. The older people who had the capacity to establish meaningful interpersonal relationships referred to support and confirmation in their relational experiences in contrast to the older people who displayed ineffective interpersonal styles. However, regardless of the interpersonal styles of the older people, the care facility was experienced as unsafe and lacking in care, which limited effective interpersonal relationships. Group activities could be implemented to assist older persons in the establishment of meaningful interpersonal relationships. Examples of these activities are board games, participating in hobbies, and learning new skills.
